# Effect of Nurses' Professionalism, Work Environment, and Communication with Health Professionals on Patient Safety Culture (AHRQ 2.0.): A Cross-Sectional Multicenter Study

**DOI:** 10.1155/2023/1591128

**Published:** 2023-08-12

**Authors:** Won Lee, Insil Jang

**Affiliations:** Department of Nursing, Chung-Ang University, Seoul, Republic of Korea

## Abstract

**Aims:**

To identify nurses' professionalism, work environment, and communication with healthcare professionals as factors influencing clinical nurses' perception of patient safety culture.

**Background:**

Patient safety is a representative indicator of the quality of nursing care. A multidisciplinary approach, including individual and organizational components, is needed to improve patient safety culture.

**Methods:**

A cross-sectional study was conducted in January 2021 involving a total of 271 nurses in six tertiary hospitals. Data were collected from participants on nurse professionalism, work environment, communication, and patient safety culture. A multiple regression model was used to analyze variables influencing patient safety culture.

**Results:**

Factors affecting patient safety culture were nursing foundations for quality of care (*β* = 0.230, *p* < 0.001), nurse manager ability, leadership, support of nurses (*β* = 0.294, *p* < 0.001), and collegial nurse-physician relations (*β* = −0.138, *p*=0.026) in the nursing work environment. Accuracy (*β* = 0.117, *p*=0.007), shift communication (*β* = 0.128, *p*=0.026), satisfaction (*β* = 0.283, *p*=0.001), and timeliness (*β* = 0.239, *p* < 0.001) of communication between healthcare professionals and nurses have a significant impact on patient safety culture. All these predictors accounted for 59% (*R*^2^) of patient safety culture awareness (*p* < 0.001).

**Conclusions:**

Enhancing patient safety culture requires a systematic and organizational approach that considers individual characteristics. Nurse managers play a crucial role in promoting patient safety by employing various communication channels and leading team education and training initiatives to foster collaboration among healthcare professionals. *Implications for Nursing Management*. The provision of patient safety education directly influences patient safety culture, and continuous education enables nurses to grow within the institution. To ensure effective communication in healthcare settings, nurse managers should prioritize shift communication, evaluate the accuracy of information exchange, and establish diverse communication channels, including social media platforms or business messengers, for internal hospital communication.

## 1. Introduction

Nurses play an important role in patient safety through nursing care, which involves direct contact with patients and includes patient surveillance, dressing and medication administration, and nursing management, such as reporting patient safety incidents, performing environmental or other unit activities, and being involved in leadership [1–3]. Patient safety is a very crucial and significant issue in nursing. To improve patient safety, a systematic, organizational approach should be considered to create a patient safety culture, which is defined as shared values, beliefs, and norms among healthcare professionals in institutions that influence their behaviors, attitudes, and actions [4]. Thus, patient safety culture not only affects patient outcomes (e.g., infection, hospital-acquired pressure ulcers, and falls) but also patient experiences [5]. It is very important to improve patient safety by identifying nurses' awareness of patient safety culture and by exploring the factors that affect patient safety culture. Several factors affect patient safety culture; in particular, professionalism, communication, and working environment are related to patient safety and safety culture [6, 7].

The nursing profession is characterized by an ongoing pursuit of knowledge, a sense of responsibility, higher education, peer accountability, autonomy, and altruism for others' wellbeing [8]. Professionalism is essential, and promoting patient safety is the key basis for achieving this [9]. Without clear policies and standards for professionalism, healthcare organizations allow unacceptable behaviors and thereby create unsafe situations [8]. Therefore, a focus on professionalism in healthcare is needed. Moreover, higher professionalism necessitates more active involvement in quality improvement actions and a greater tendency to report medical errors [10]. Thus, the relationship between influencing factors of professionalism and patient safety culture needs to be investigated.

As previously established, communication and patient safety are of great importance. Latent conditions such as inter- or intrateam communication problems have been shown to increase the risk of medication errors, as noted by Burgener [11]. Communication between healthcare professionals and patients has been identified as the main contributory factor for adverse events arising from negligence in nursing care and can lead to delayed care, physical harm, and dissatisfaction [12, 13]. Therefore, effective communication between healthcare professionals is important for patient safety and quality of care [14]. Trust-based open communication that enables the expression of safety issues and concerns is known to be one of the dimensions of safety culture; therefore, the relationship between communication and safety culture needs to be explored [15].

A healthy work environment enables safe, empowered, and satisfactory work by nurses [16] and influences nursing outcomes, including psychological health, job performance, and job satisfaction [17]. The nursing work environment influences and is related to patient safety; improved work environments have been shown to be associated with increased care quality, patient satisfaction, and a more positive safety culture in pediatric care settings [18]. According to Rainbow et al. [1], the adverse event reporting practices of nurses were influenced by their perceptions of the work environment.

The measurement and assessment of patient safety culture could help to ascertain the staff's attitudes and beliefs with regard to patient safety [19]. Therefore, determining the extent of patient safety culture is the first step toward promoting a positive safety culture, and employees' safety culture perceptions should be evaluated to improve patient safety as well as patient outcomes [19]. The Hospital Survey on Patient Safety Culture, developed by the Agency for Healthcare Research and Quality (AHRQ), is the most widely used tool for measuring patient safety culture [15], and several questionnaire items have been modified, such as by rewording complex survey items, by adding a response option, and by shifting to a “just culture” to assess the response to an error; in 2019, the AHRQ version 2.0 was issued.

The relationships among professionalism as an individual aspect, communication with health professionals as a team factor, and nursing work environment as a structural aspect of patient safety culture have not been investigated. When measuring patient safety culture, it is important to take into account unique regional characteristics, as highlighted by Waterson et al. [20], and few studies using the AHRQ version 2.0 have been performed. Thus, research into the relationships among individual, team, and structural aspects of patient safety culture is needed. Through this study, we intend to provide basic data for creating a patient safety culture and improving patient safety by confirming patient safety culture awareness using the AHRQ version 2.0 tool and exploring the factors that affect it.

This study aimed to identify factors that affect clinical nurses' perception of patient safety culture. The study-specific objectives were as follows: (1) to determine each participant's general characteristics, professionalism, nursing work environment, and communication with healthcare professionals as these factors relate to patient safety culture; (2) to examine the correlation of professionalism, nursing work environment, and communication with healthcare professionals with patient safety culture; and (3) to identify factors that influence nurses' perception of patient safety culture.

## 2. Method

### 2.1. Study Design

In 2021, a cross-sectional survey was carried out across multiple centers, and the findings were reported in adherence with the STROBE guidelines [21] (supplementary file 1).

### 2.2. Participants

The study aimed to recruit full-time registered nurses with a minimum of one year of experience working in various units, such as general wards, outpatient departments, and intensive care units, among others, across six tertiary general hospitals in South Korea. Participation in the study was voluntary, and nurses who were not involved in patient care were excluded from the study. The sample size needed for the study was determined using the G-power 3.1 program, with an *α* of 0.05, an effect size of 0.15 (medium), and a power (1 − *β*) of 0.95, based on a linear multiple regression analysis, which indicated that 189 participants were required. To account for a possible attrition rate of 30%, data were collected from more than 270 nurses.

### 2.3. Instruments with Validity and Reliability

#### 2.3.1. Demographic and Work-Related Questionnaire

Each participant's sex, age, education, total years of clinical experience, number of years of clinical experience in the current department, work unit, job position, type of duty, attended patient safety-related education, patient safety incident experience, types of patient safety incident experience, and classification of patient safety incident were collected.

#### 2.3.2. Professionalism

To evaluate the participants' professionalism, a modified version of Hall's Professionalism Inventory (HPI) Scale [22] was utilized. Snizek [23] condensed the original 50-item HPI into 25 items, which were translated into Korean by Kim and Baek [24]. The 25-item Korean version is scored on a five-point Likert scale, with five items for each of the five subdimensions: professional community affiliation, public service, autonomy, self-regulation, and sense of calling. The tool's internal reliability was measured using Cronbach's *α*, which was found to be 0.82 in the study conducted by Kim and Baek [24] and 0.73 in this study.

#### 2.3.3. Nursing Work Environment

The nursing work environment is an organizational feature of a workplace that promotes or hinders professional nursing practice and includes all aspects of the physical environment perceived by nurses, interactions among organizational members, organizational aspects that affect the contents and methods of nurses' work performance, and policy [25]. The Korean version of the Practice Environment Scale of Nursing Work Index (K-PES-NWI), which was verified for reliability and validity after it was developed by Lake and Friese [26] and translated by Cho et al. [27], was used with the author's approval. This tool consists of the following five subdimensions with 29 items: nursing foundations for quality of care; nurse manager ability, leadership, and support of nurses; nurse participation in hospital affairs; collegial nurse-physician relations; and staffing and resource adequacy. Participants rated these items on a four-point Likert scale from one (strongly disagree) to four (strongly agree), with higher scores denoting a more positive nursing work environment. In the study by Cho et al. [27], Cronbach's *⍺* was 0.93; in this study, Cronbach's *⍺* was 0.94.

#### 2.3.4. Nurses' Communication with Health Professionals

Communication between nurses and health professionals indicates that nurses express their opinions in an open atmosphere for patient safety, deliver accurate information in a timely manner, understand cross-professional perspectives, and communicate effectively during shift transfer [28]. The 30-item ICU Nurse-Physician Questionnaire, which includes 16, 11, and three items for nurse-doctor, nurse-nurse, and general communication, respectively, was developed by Shortell et al. [29]; the Korean version was translated and validated by Cho et al. [28]; this tool was used and scored using a five-point Likert scale (score range: 30–150); negative response questions are inversely converted. The five subdimensions include openness, accuracy, understanding, shift communication, satisfaction, and timeliness. A higher score indicates more positive perception of communication between health professionals. The overall value of Cronbach's *α* in the tool development study was 0.89, and that of each subdimension was 0.59–0.89. Cronbach's *⍺* in this study was 0.93.

#### 2.3.5. Patient Safety Culture

The Korean version of the HSOPSC version 2.0, which was validated by the Agency for Healthcare Research and Quality [30], was used. This tool consists of 31 items in the following 12 dimensions: D1: teamwork, D2: staffing and work pace, D3: organizational learning and continuous improvement, D4: response to error, D5: supervisor, manager, or clinical leader support for patient safety, D6: communication about error, D7: communication openness, D8: reporting patient safety events, D9: hospital management support for patient safety, D10: handoffs and information exchange, D11: number of events reported, and D12: patient safety grade. The responses are measured on a five-point Likert scale, with scores varying for each item. To analyze the scale, negative questions were converted into their positive equivalents. Responses were categorized into three groups: negative (completely disagree/never, disagree/rarely), neutral (neither agree nor disagree/sometimes), and positive (agree/almost always, completely agree/always). This categorization was used to classify the items and dimensions of the instrument as strengths and areas for improvement. Cronbach's *⍺* for the 10 scales (D1–D10) of the HSOPSC 2.0 ranged from 0.67 to 0.89 in the U.S. study [31]. In this study, the overall value of Cronbach's *α* was 0.84 and ranged from 0.62 to 0.83 for the various subdimensions.

### 2.4. Data Collection and Ethical Considerations

The online questionnaire in Google Forms was completed by clinical nurses (*N* = 287) at six tertiary general hospitals in January 2022. After excluding 16 incomplete questionnaires, data from 271 questionnaires were analyzed. The researcher provided an information sheet to potential participants before the study to explain the study's purpose and assure them that their responses would be kept confidential and used only for research purposes. Participants were required to provide consent by signing the first page of the questionnaire after understanding the purpose of the study. This study was approved by an appropriate institutional review board (IRB No. 2021-1551), and the investigation conformed to the principles outlined in the 1964 Declaration of Helsinki.

### 2.5. Data Analysis

Data were analyzed with SPSS version 25.0 for Windows by first checking for outliers and missing responses, and then excluding incomplete responses. Demographic characteristics were analyzed using descriptive statistics, including frequencies, percentage, means, and standard deviations. Internal consistency coefficients (Cronbach's *α*) were calculated. An independent *t*-test and one-way ANOVA were performed to assess patient safety culture according to the participants' demographic and work-related characteristics. Correlations between patient safety culture awareness and nurses' professionalism, work environment, and communication with health professionals were ascertained using Pearson's correlation. Linear multiple regression analysis, including the Durbin–Watson test, was used to identify factors that influenced the participants' patient safety culture awareness; *p* < 0.05 was considered statistically significant.

## 3. Results

### 3.1. Demographic and Work-Related Characteristics

The participants' demographic and work-related characteristics are shown in Table 1. Of the 271 participants, 97.8% were female, 43.5% were in the 30–39-year-old age group, 64.9% had bachelor's degrees, 59.0% had ≥10 years of clinical experience, 70.5% were staff registered nurses, and 59.0% were shift workers. The majority of the participants had received patient safety-related education (98.5%), experienced patient safety incidents (91.1%), and experienced near miss (59.4%). Patient safety culture according to demographic and work-related characteristics showedsignificant differences by patient safety related education completion (*U* = 2.021, *p* = 0.041) and by the nurse's position (*F* = 7.011, *p* < 0.001).

### 3.2. Descriptive Statistics

Nurses' mean scores (SD) for professionalism, work environment, and communication with health professionals were 3.18 (SD 0.35, range 2.08–3.96), 2.51 (SD 0.50, range 1.21–3.69), and 3.13 (SD 0.55, range 1.37–4.80), respectively (Table 2). The total mean score of the hospital survey on patient safety culture was 3.42 (SD 0.47), with the highest mean score of 3.82 (SD 0.65) for teamwork and the lowest mean score of 2.85 (SD 0.69) for staffing and workplace (Table 3). Table 3 presents the percentages of positive responses and mean scores for patient safety culture.

### 3.3. Correlation of Nurses' Professionalism, Work Environment, and Communication with Health Professionals with Patient Safety Culture

The nursing professionalism subdimensions that were positively correlated with the perception of patient safety culture included public service (*r* = 0.181, *p* < 0.001) and a sense of calling (*r* = 0.317, *p* < 0.001). All nurses' work environment dimensions (*r* = 0.601, *p* < 0.001) and communication with health professionals (*r* = 0.588, *p* < 0.001) were positively correlated with patient safety culture (Table 4).

### 3.4. Multiple Regression Analysis to Identify Factors That Influence Patient Safety Culture

Table 5 shows the results of the detailed multiple regression analysis of areas of nurses' professionalism, work environment, and communication with health professionals as factors that influence nurses' perception of patient safety culture. The Durbin–Watson value for the residual analysis of each variable was 2.043, which suggests that the variables are independent of each other, and the variance expansion index (VIF) values were all less than 10, indicating no multicollinearity, and the regression model was statistically significant (*F* = 26.53, *p* < 0.001). Factors that significantly influenced the subjects' perception of patient safety culture were nursing foundations for quality of care (*β* = 0.230, *p*  <  0.001), nurse manager ability, leadership, and support of nurses (*β* = 0.294, *p*  <  0.001), and collegial nurse-physician relations (*β* = −0.138, *p*=0.026) in the nursing work environment. Accuracy (*β* = 0.117, *p*=0.007), shift communication (*β* = 0.128, *p*=0.026), satisfaction (*β* = 0.283, *p*=0.001), and timeliness (*β* = 0.239, *p*  <  0.001) were identified as important factors related to communication between health professionals and nurses. These influencing factors accounted for 59% of the patient safety culture perceptions (Table 5).

## 4. Discussion

This study examines the relationship between nurses' professionalism, communication with health professionals, work environment, and their perceptions of patient safety culture, considering individual, team, and structural aspects. The findings provide valuable insights for nursing management in fostering a safety culture that encompasses these multidimensional factors.

Although the percentage of respondents who received patient safety education among individual factors was relatively high, the perception of patient safety culture showed a significant difference according to whether or not they received patient safety education. Amiri et al. [32] found that educational empowerment programs, including patient safety, patient safety culture, speak out, and the skills of Team Strategies and Tools to Enhance Performance and Patient Safety (TeamSTEPPS) programs, improved patient safety culture. In addition, a study among physicians and nurses about the patient safety culture during the adoption of patient safety policy showed that continuous patient safety education for accreditation evaluation positively influenced the creation of a patient safety culture [33]. Patient safety education is important not only to disseminate knowledge but also to facilitate emphasizing patient safety first and to reinforce the shared beliefs of members; therefore, continuous patient safety-related education should be imparted. Among the various individual factors, although their influence was not significant, the factors that affected patient safety culture among the dimensions of professionalism were public service and a sense of calling. More positive professionalism has been shown to increase awareness of patient safety culture, and greater recognition of the professionalism of nursing service and a higher sense of calling have been shown to increase recognition of the importance of patient safety in nursing practice [6, 34, 35]. This study confirmed the abovementioned correlation, despite the lack of a statistically significant influence on the perception of patient safety culture. Few studies of the effects of professionalism on patient safety culture after the coronavirus disease pandemic have been conducted; thus, studies are needed to determine whether the relevance is weakening due to changes in the medical environment. Furthermore, evaluation of changes in the attributes of nursing professionalism is necessary to evaluate various tools to measure professionalism and identify tool validity to ascertain their suitability for the current health environment and clinical situation [36].

All subcategories related to nurses' communication with health professionals were correlated with patient safety culture, and accuracy, shift communication, satisfaction, and timeliness affected their perception of patient safety culture. Accuracy and timeliness are very important components of the communication process, and the belief in timely communication of accurate information among healthcare professionals and satisfaction with communication are very important influencing factors for creating a positive patient safety culture [37]. Effective communication during shift handover is critical due to the continuity of care and 24/7 work environment. Recent studies have emphasized several key factors that influence the effectiveness of shift handover, including the structure and content of handover protocols, the use of technology to support communication, and the impact of organizational culture on communication practices [38, 39]. The complexity of the handover process varies depending on the roles and stances of incoming versus outgoing nurses and is influenced by individual and organizational factors. Therefore, nursing managers should particularly evaluate these factors and manage intradepartmental communication. Accurate communication among healthcare professionals is important for ensuring the patients' perceived safety during hospitalization [40]; thus, efforts to improve the accuracy and timeliness of communication is important to improve patient safety culture, including patients' perceptions of safety.

In terms of the structured aspect, nurses' work environments were correlated with their perception of patient safety culture. Moreover, a foundation for quality of care, support for nurses by managers and leaders who have the ability, and good nurse-physician relations were significantly associated with patient safety culture. The foundation of nursing quality is a key consideration in nursing management, and nurse managers should secure qualitative continuity of nursing by assigning appropriate staff nurses to patients and thereby support nurses [41]. Educational programs that can develop nurses' competency and careers should be continuously provided at the institutional level or through associations to provide high-quality nursing care to patients. Teamwork is an important aspect of patient safety culture. Wami et al. [42] found that team collaboration with support and respect among professionals is essential in safety culture. Previous studies have shown that a cooperative relationship between nurses and physicians was important for nurses' job satisfaction, patient safety, and safety culture [7]. Recent studies have shown that training focused on teamwork and communication training can potentially improve safety culture and positively impact patient outcomes, and has been considered a promising strategy for reducing medical errors and adverse events [43, 44]. The importance of effective communication should be acknowledged for improving nurse-physician collaboration; therefore, a foundation of proper communication including interprofessional communication education programs for nurses and physicians beginning when they are undergraduate students is needed [45]. Nurses' work environment affects their physical and psychological health and thereby affects patient outcomes [7]. To cultivate a culture of safety in healthcare, it is essential to provide nurses with access to career development programs, as well as to demonstrate strong leadership and support from managers. Additionally, fostering collaborative relationships and promoting teamwork across different healthcare occupations can further enhance patient safety.

## 5. Conclusion

This study was conducted to identify factors that affect nurses' contribution to patient safety culture, and these included the following: nursing foundation for care quality; nurse manager's ability, leadership, and support of nurses; and collegial nurse-physician relations in nurses' work environments with the subfactors: accuracy, shift communication, satisfaction, and timeliness of health professional-nurse communication. To improve patient safety culture, a systematic, organizational approach with individualized characteristics is essential, and this will facilitate institutional improvement strategies for transparent communication and activation of various communication channels through social media platforms or business messengers for internal communication of the hospital. Furthermore, patient safety education using a team approach and training for collaborative teamwork among healthcare professionals is the cornerstone for improving nursing quality and establishing a patient safety culture.

## 6. Implication for Nursing Management

The study findings emphasize the importance of a systematic and organizational approach in establishing a patient safety culture. Predictors of patient safety culture identified include accuracy, shift communication, satisfaction, and timeliness in nurses' communication with healthcare professionals. These findings have significant clinical implications. Unit managers are encouraged to evaluate communication status and patterns, focusing on accuracy, timeliness, and nurses' satisfaction. Furthermore, it is recommended to quantify the specific information exchanged during shift communication, enabling nurses to utilize tools and strategies to enhance communication.

Within the nursing work environment, supportive and competent managers and leaders, as well as positive relationships with physicians, were found to influence patient safety culture. Establishing career development programs to foster nursing leadership skills and cultivating collaborative relationships with physicians can contribute to creating an organizational culture that prioritizes safety. Additionally, nursing leaders should provide institutional-level continuous education on patient safety and nursing care, laying the groundwork for nursing quality improvement initiatives. Therefore, nurse managers play a crucial role in creating a healthy nurse work environment by fostering a positive culture and ensuring effective communication, high-quality nursing care, and teamwork. They are responsible for providing guidance and support to nursing staff, promoting a safe and supportive atmosphere that values open communication, collaboration, and mutual respect. These efforts further contribute to enhancing patient safety culture and overall healthcare outcomes.

## 7. Limitations

Although the sampling was randomized, the study only included nurses from six tertiary hospitals, limiting the generalizability and representativeness of the results. It is necessary to replicate the findings with larger multicenter studies. Additionally, the results were based on self-reported measures, indicating that objective indicators, such as behavioral measures, should be included in future studies.

## Figures and Tables

**Table 1:** Demographic and work-related characteristics of participants and differences in patient safety culture by demographic and work-related characteristics (*N* = 271).

Variables	Categories	Frequency (%)	Mean ± SD	Patient safety culture	
				Mean ± SD	*t* or *F*(*p*) Scheffe
Sex	Male	6 (2.2)		3.58 ± 0.58	0.847 (0.398)
	Female	265 (97.8)		3.41 ± 0.47	

Age (years)	20–29	81 (29.9)	35.19 ± 7.32	3.46 ± 0.42	3.307 (0.058)
	30–39	118 (43.5)		3.34 ± 0.46	
	≥40	72 (26.6)		3.50 ± 0.54	

Education	Associate degree	14 (5.2)		3.23 ± 0.55	2.645 (0.050)
	Baccalaureate	176 (64.9)		3.39 ± 0.42	
	Master's degree or higher	81 (29.9)		3.51 ± 0.55	

Total years of clinical experience	1∼5	67 (24.7)	12.08 ± 7.67	3.47 ± 0.52	1.024 (0.361)
	5∼10	44 (16.2)		3.34 ± 0.53	
	≥10	160 (59.0)		3.42 ± 0.47	

Number of years of clinical experience in the current department	<1	24 (8.8)	5.74 ± 5.23	3.42 ± 0.54	0.506 (0.604)
	1∼3	79 (29.2)		3.43 ± 0.49	
	3∼5	60 (22.1)		3.37 ± 0.51	
	≥5	108 (39.9)		3.44 ± 0.44	

Work unit	Medical and surgical	139 (51.3)		3.47 ± 0.46	1.721 (0.146)
	Intensive care	34 (12.5)		3.33 ± 0.47	
	Operating room	12 (4.4)		3.20 ± 0.43	
	Outpatient	51 (18.8)		3.35 ± 0.53	
	Others	35 (12.9)		3.48 ± 0.42	

Job position	Staff RN^a^	191 (70.5)		3.37 ± 0.41	7.011 (<0.001)^a<c<b^
	Charge nurse or higher^b^	28 (10.3)		3.57 ± 0.54	
	Others (CNS, coordinator, etc.)^c^	52 (19.2)		3.49 ± 0.47	

Type of duty	Shift worker	160 (59.0)		3.37 ± 0.45	−1.946 (0.053)
	Nonshift worker	111 (41.0)		3.48 ± 0.51	

Attended patient safety-related education	Yes	267 (98.5)		3.42 ± 0.47	2.021 (0.041)^*∗*^
	No	4 (1.5)		2.94 ± 0.41	

Patient safety incident experience	Yes	247 (91.1)		3.43 ± 0.48	1.115 (0.265)^*∗*^
	No	24 (8.9)		3.31 ± 0.40	

Types of patient safety incident experience	Direct	146 (53.9)		3.44 ± 0.47	0.432 (0.666)
	Indirect	101 (37.3)		3.41 ± 0.50	

Classification of patient safety incident	Near miss	161 (59.4)		3.43 ± 0.48	0.679 (0.566)
	Adverse event	75 (27.7)		3.44 ± 0.48	
	Sentinel event	11 (8.9)		3.31 ± 0.54	

^*∗*^Mann–Whitney *U* test. RN = registered nurse; CNS = clinical nurse specialist. Superscripts a,b, and c in job position are Scheffe test contents and are marked in the categories.

**Table 2:** Professionalism, nursing work environment, and nurses' communication with health professionals (*N* = 271).

Variables	Range	Mean ± SD	Min	Max
Professionalism	1∼5	3.18 ± 0.35	2.08	3.96
Professional community affiliation		2.95 ± 0.55	1.20	4.60
Public service		3.43 ± 0.46	2.00	4.60
Autonomy		3.05 ± 0.44	2.00	4.60
Self-regulation		3.26 ± 0.47	1.40	4.80
Sense of calling		3.21 ± 0.63	1.20	5.00

Nursing work environment	1∼4	2.51 ± 0.50	1.21	3.69
Nursing foundations for quality of care		2.71 ± 0.55	1.00	4.00
Nurse manager ability, leadership, and support of nurses		2.65 ± 0.63	1.00	4.00
Nurse participation in hospital affairs		2.44 ± 0.58	1.00	4.00
Collegial nurse-physician relations		2.55 ± 0.70	1.00	4.00
Staffing and resource adequacy		2.06 ± 0.61	1.00	3.75

Nurses' communication with health professionals	1∼5	3.13 ± 0.55	1.37	4.80
Openness		3.22 ± 0.72	1.00	5.00
Accuracy		3.06 ± 0.62	1.29	4.71
Understanding		2.76 ± 0.81	1.00	5.00
Shift communication		3.48 ± 0.77	1.00	5.00
Satisfaction		3.18 ± 0.80	1.00	5.00
Timeliness		3.76 ± 0.64	1.33	5.00

**Table 3:** HSOPSC 2.0 means and percentages of positive responses (*N* = 271).

Variables	Range	Positive responses (%)	Mean ± SD	Min	Max
			Frequency (%)		
Patient safety culture	1∼5	51.20	3.42 ± 0.47	1.35	4.61
Teamwork		70.80	3.82 ± 0.65	1.33	5.00
Staffing and work place		29.76	2.85 ± 0.69	1.00	4.67
Organizational learning-continuous improvement		50.80	3.44 ± 0.67	1.00	5.00
Response to error		40.35	3.15 ± 0.73	1.00	5.00
Supervisor, manager, or clinical leader support for patient safety		69.47	3.80 ± 0.67	1.00	5.00
Communication about error		57.11	3.69 ± 0.79	1.33	5.00
Communication openness		49.06	3.39 ± 0.75	1.00	5.00
Reporting patient safety events		51.78	3.47 ± 0.82	1.00	5.00
Hospital management support for patient safety		39.69	3.19 ± 0.75	1.00	5.00
Handoffs and information exchange		57.69	3.54 ± 0.64	1.67	5.00

Number of events reported during 1 year (single item measure)	No		131 (48.3)		
	1∼2		95 (35.1)		
	3∼5		31 (11.4)		
	6∼10		7 (2.6)		
	≥11		7 (2.6)		

Patient safety grade (single item measure)	1∼5		3.49 ± 0.78	1.00	5.00

**Table 4:** Correlation among professionalism, nursing work environment, nurses' communication with health professionals, and patient safety culture (*N* = 271).

Variable	*r* (*p*)																			
	PF	1	2	3	4	5	NE	6	7	8	9	10	NC	11	12	13	14	15	16	PSC
Professionalism	1																			
(1) Professional community affiliation	0.736^*∗∗*^	1																		
(2) Public service	0.710^*∗∗*^	0.366^*∗∗*^	1																	
(3) Autonomy	0.610^*∗∗*^	0.310^*∗∗*^	0.292^*∗∗*^	1																
(4) Self-regulation	0.645^*∗∗*^	0.322^*∗∗*^	0.402^*∗∗*^	0.407^*∗∗*^	1															
(5) Sense of calling	0.723^*∗∗*^	0.460^*∗∗*^	0.427^*∗∗*^	0.220^*∗∗*^	0.200^*∗∗*^	1														
Nursing work environment	0.060	0.058	0.069	−0.139^*∗*^	−0.204^*∗∗*^	0.312^*∗∗*^	1													
(6) Nursing foundations for quality of care	0.076	0.086	0.133^*∗*^	−0.161^*∗∗*^	−0.200^*∗∗*^	0.299^*∗∗*^	0.886^*∗∗*^	1												
(7) Nurse manager ability, leadership, and support of nurses	0.073	0.064	0.129^*∗*^	−0.149^*∗∗*^	−0.149^*∗*^	0.267^*∗∗*^	0.789^*∗∗*^	0.628^*∗∗*^	1											
(8) Nurse participation in hospital affairs	0.003	0.014	−0.002	−0.134^*∗*^	−0.213^*∗∗*^	0.247^*∗∗*^	0.925^*∗∗*^	0.750^*∗∗*^	0.675^*∗∗*^	1										
(9) Collegial nurse-physician relations	0.078	0.028	0.079	−0.043	−0.100	0.238^*∗∗*^	0.755^*∗∗*^	0.607^*∗∗*^	0.519^*∗∗*^	0.629^*∗∗*^	1									
(10) Staffing and resource adequacy	0.051	0.049	−0.058	−0.022	−0.111	0.239^*∗∗*^	0.703^*∗∗*^	0.463^*∗∗*^	0.492^*∗∗*^	0.602^*∗∗*^	0.516^*∗∗*^	1								
Nurses' communication with health professionals	0.190^*∗∗*^	0.074	0.149^*∗*^	−0.009	−0.067	0.411^*∗∗*^	0.543^*∗∗*^	0.488^*∗∗*^	0.417^*∗∗*^	0.450^*∗∗*^	0.562^*∗∗*^	0.362^*∗∗*^	1							
(11) Openness	0.278^*∗∗*^	0.169^*∗∗*^	0.206^*∗∗*^	0.027	0.060	0.414^*∗∗*^	0.475^*∗∗*^	0.427^*∗∗*^	0.385^*∗∗*^	0.384^*∗∗*^	0.509^*∗∗*^	0.301^*∗∗*^	0.897^*∗∗*^	1						
(12) Accuracy	−0.200^*∗∗*^	−0.159^*∗∗*^	−0.072	−0.297^*∗∗*^	−0.328^*∗∗*^	0.082	0.296^*∗∗*^	0.284^*∗∗*^	0.274^*∗∗*^	0.224^*∗∗*^	0.254^*∗∗*^	0.200^*∗∗*^	0.480^*∗∗*^	0.172^*∗∗*^	1					
(13) Understanding	0.195^*∗∗*^	0.093	0.092	0.117	−0.041	0.344^*∗∗*^	0.467^*∗∗*^	0.378^*∗∗*^	0.309^*∗∗*^	0.397^*∗∗*^	0.559^*∗∗*^	0.358^*∗∗*^	0.871^*∗∗*^	0.749^*∗∗*^	0.242^*∗∗*^	1				
(14) Shift communication	0.178^*∗∗*^	0.015	0.157^*∗∗*^	0.023	0.050	0.314^*∗∗*^	0.268^*∗∗*^	0.275^*∗∗*^	0.229^*∗∗*^	0.247^*∗∗*^	0.165^*∗∗*^	0.128^*∗∗*^	0.604^*∗∗*^	0.590^*∗∗*^	0.194^*∗∗*^	0.332^*∗∗*^	1			
(15) Satisfaction	0.235^*∗∗*^	0.094	0.154^*∗∗*^	0.109	0.043	0.350^*∗∗*^	0.453^*∗∗*^	0.395^*∗∗*^	0.323^*∗∗*^	0.381^*∗∗*^	0.510^*∗∗*^	0.305^*∗∗*^	0.872^*∗∗*^	0.832^*∗∗*^	0.252^*∗∗*^	0.754^*∗∗*^	0.591^*∗∗*^	1		
(16) Timeliness	0.237^*∗∗*^	0.077	0.248^*∗∗*^	0.005	0.046	0.372^*∗∗*^	0.366^*∗∗*^	0.409^*∗∗*^	0.282^*∗∗*^	0.319^*∗∗*^	0.244^*∗∗*^	0.162^*∗∗*^	0.590^*∗∗*^	0.556^*∗∗*^	0.139^*∗∗*^	0.354^*∗∗*^	0.525^*∗∗*^	0.511^*∗∗*^	1	
Patient safety culture	0.118	0.054	0.181^*∗∗*^	−0.088	−0.124	0.317^*∗∗*^	0.601^*∗∗*^	0.584^*∗∗*^	0.575^*∗∗*^	0.499^*∗∗*^	0.404^*∗∗*^	0.357^*∗∗*^	0.588^*∗∗*^	0.526^*∗∗*^	0.324^*∗∗*^	0.357^*∗∗*^	0.496^*∗∗*^	0.545^*∗∗*^	0.559^*∗∗*^	1

PF, professionalism; NE, nursing work environment; NC, nurses' communication with health professionals; PSC, patient safety culture. ^*∗*^Correlation is significant at the 0.05 level (two-tailed). ^*∗∗*^Correlation is significant at the 0.01 level (two-tailed).

**Table 5:** Multiple regression analysis to identify the factors influencing patient safety culture (*N* = 271).

Variables		*B*	*β*	*t*	*p*	VIF
Work-related characteristics	(Constant)	1.275		4.820	<0.001	
	Job position	0.009	0.022	0.506	0.613	1.239
	Attended patient safety-related education	−0.204	−0.052	−1.280	0.202	1.074

Professionalism	Public service	−0.053	−0.052	−1.115	0.266	1.396
	Sense of calling	0.016	0.022	0.446	0.656	1.578

Nursing work environment	Nursing foundations for quality of care	0.199	0.230	3.391	<0.001	2.998
	Nurse manager ability, leadership, and support of nurses	0.222	0.294	5.100	<0.001	2.172
	Nurse participation in hospital affairs	−0.062	−0.075	−1.037	0.301	3.441
	Collegial nurse-physician relations	−0.093	−0.138	−2.244	0.026	2.468
	Staffing and resource adequacy	0.039	0.050	0.951	0.342	1.793

Nurses' communication with health professionals	Openness	0.006	0.010	0.112	0.911	4.905
	Accuracy	0.090	0.117	2.700	0.007	1.217
	Understanding	−0.045	−0.077	−1.096	0.274	3.254
	Shift communication	0.079	0.128	2.243	0.026	2.114
	Satisfaction	0.168	0.283	3.460	0.001	4.359
	Timeliness	0.176	0.239	4.528	<0.001	1.823

*R* ^2^ = 0.61, Adj *R*^2^ = 0.59, and *F* (*p*)=26.53 (<0.001)						

## Data Availability

The data that support the findings of this study are available from the corresponding author upon reasonable request.

## Supplementary Materials

Supplementary Materials Supplementary File 1: the STROBE checklist.
